# Editorial on the Special Issue: “Advances in Xerogels: From Design to Applications”

**DOI:** 10.3390/gels9060446

**Published:** 2023-05-27

**Authors:** Andrea Fiorati, Francesco Caridi, Giuseppe Paladini

**Affiliations:** 1 Department of Chemistry, Materials, and Chemical Engineering “G. Natta” INSTM Local Unit, Politecnico di Milano, 20131 Milan, Italy; 2 Department of Mathematical and Computer Sciences, Physical Sciences and Earth Sciences, University of Messina, 98166 Messina, Italy; fcaridi@unime.it; 3 Department of Physics and Astronomy “Ettore Majorana”, University of Catania, 95123 Catania, Italy; giuseppe.paladini@dfa.unict.it

Xerogels are solid materials derived from gels which consist of interconnected particles or polymers dispersed in a liquid. This gel structure is subjected to a drying process (e.g., slow evaporation or freeze-drying), resulting in the removal of the liquid phase, leaving behind a solid material. During this drying process, the liquid is extracted from the gel while attempting to preserve its original shape and structure as much as possible. Although some shrinkage may occur, xerogels exhibit unique properties such as high porosity and a large surface area due to the interconnected network of pores formed during drying. These characteristics make xerogels valuable in a wide range of applications, including catalysts, adsorbents, sensors, membranes, and drug delivery systems. The field of materials science and engineering has seen a surge of interest in the development of innovative xerogels, leading to a focus on their design for various applications. With the ability to conveniently control their structures and morphologies during synthesis, xerogels have become a subject of great significance within the scientific community.

Taking these factors into account, the objective of this *Gels* Special Issue is to assemble high-quality papers that showcase recent progress and discoveries in xerogel science, encompassing design and practical utilization. We sought original contributions exploring traditional and non-traditional approaches to synthesizing and characterizing xerogels. The aim is to deepen our understanding of the fundamental and applied aspects of various organic and inorganic xerogel-like materials.

The recent advancements concerning the role of biopolymer-based xerogels in biomedical applications were thoroughly discussed by Khalil and colleagues. Through their systematic literature review, the authors provide insights into the biological properties of xerogels that make them suitable materials for various biomedical applications, including drug delivery, wound healing and dressing, tissue scaffolding, and biosensing [1].

Furthermore, xerogels are widely utilized for the adsorption of ions and metals. Putz et al. [2] reported the synthesis of ordered mesoporous silica materials capable of acting as sorbents for environmental remediation, specifically targeting Cu(II) and Pb(II). Xerogels’ metal adsorption properties can be effectively harnessed to create efficient heterogeneous catalysts. For instance, Riva et al. [3] successfully catalyzed the Suzuki–Miyaura coupling reaction using Pd-loaded cellulose nanosponge as a heterogeneous catalyst. Additionally, Mahy et al. [4] described the preparation of mono- and bimetallic catalysts based on Fe, Ni, and Pd supported on silica through a sol–gel co-gelation process, resulting in porous materials with surface areas ranging from 100 to 400 m^2^/g.

Although silica xerogels are widely recognized as the most commonly used type, it is known that metal oxides such as Al_2_O_3_, SiO_2_, TiO_2_, and CeO_2_, which possess large specific surfaces and thermal stability, can serve as catalyst supports. Within our special issue, we feature the research of Romanczuk-Ruszuk and colleagues, who synthesized binary xerogel systems of Sr/Al using the sol–gel technique. These systems incorporated a metallic strontium precursor and were evaluated as supports for platinum catalysts. The researchers successfully achieved highly dispersed and stable strontium carbonate phases, leading to a remarkable dispersion (42–50%) of platinum nanoparticles [5].

The versatility of sol–gel approaches can be exploited to trap ions (as reported before) or molecules and macromolecules such as proteins to confer the final materials’ specific properties. For the sake of an example, Morosanova and colleagues reported the synthesis of a promising biosensor development by incorporating enzymes (horseradish peroxidase and mushroom tyrosinase) and crude banana extract in a silane-based structure to create optical biosensors for hydrogen peroxide in the range of 0.2–3.5 mM [6].

The classic sol–gel process which involves hydrolysis reactions of (semi)metal alkoxides is a well-established method for creating organic–inorganic hybrid materials. However, alternative approaches utilizing non-aqueous or non-hydrolytic systems have also been investigated. In this context, Krupinski and colleagues presented a novel approach utilizing element chlorides and silylated precursors to produce “bridged” or “linearly expanded” silicas and silsesquioxanes through a non-hydrolytic sol–gel method. This study demonstrates that the non-hydrolytic approach to hybrid materials can be extended to other silylated precursors, as long as the reactivity of the corresponding chlorine compound is adequate [7].

The responsibility of porous materials toward external stimuli is crucial for the obtainment of smart innovative materials, and the chemistry of N-oxide moieties offers a wide range of possibilities. In this context, Trofimov and co-workers report the synthesis of 4-dialkylamino-2,5-dihydroimidazol-1-oxyls with moieties at position 2 and at the exocyclic nitrogen able to act as pH-Sensitive spin labels [8], while Jayabhavan and colleagues study the stimuli-responsivity supramolecular gels based on pyridyl-N-oxide amides [9].

Due to their versatility, xerogels and porous materials are becoming of great interest to scientists who focus their attention on food science and food packaging. Dried porous materials based on plant proteins have gained significant attention as potential sustainable food ingredients. However, plant proteins exhibit weaker gelling properties compared to animal proteins. To enhance plant protein gelling, optimization of gelation conditions involving protein concentration, pH, and ionic strength is necessary. De Berardinis and colleagues conducted a systematic study to investigate the impact of these factors on the gelation behavior of soy and pea protein isolates. The findings were used to create a map that identifies the gelation conditions for modulating the rheological properties of soy and pea protein hydrogels, with potential applications in the production of xerogels, cryogels, and aerogels [10]. Innovation in intelligent food packaging materials holds promise for enhancing food safety, quality, and control. Researchers are exploring the combination of biodegradable semi-synthetic polymers with natural polymers and additives to improve material functionality. In line with this, Vlad-Bubulac and colleagues developed composite films by casting a solution containing specific mass ratios of poly(vinyl alcohol) and chitosan as the polymeric matrix, supplemented with TiO_2_ nanoparticles and a polyphosphonate as reinforcing additives. The favorable outcomes regarding precursor homogeneity, film quality, antimicrobial activity, and cytocompatibility demonstrate the potential suitability of these films for food packaging applications [11].

Xerogels offer unique properties for diverse applications, including catalysts, adsorbents, sensors, and drug delivery systems. Biopolymer-based xerogels show promise in biomedical applications, while metal oxides expand catalyst support options. Non-hydrolytic sol–gel approaches and additives enable hybrid materials. Intelligent food packaging and improved plant protein gelling are active areas of research. Composite films combining biodegradable polymers, natural polymers, and additives hold potential for food packaging. Exciting advancements in xerogel science drive innovation stimulating the interest of the scientific community.
